# Research on mechanism and control technique of floor heave in deep soft rock roadway at Banji Coal Mine

**DOI:** 10.1038/s41598-026-48221-y

**Published:** 2026-04-16

**Authors:** Xun Zhang, Laiwang Jing, Hailong Dong, Heng He, Jiang Lv, Maoru Sun, Yuanfeng Fan

**Affiliations:** 1 Banji Coal Mine, Xinji Energy Company Limited, National Coal Group Corporation, Bozhou, 236744 Anhui China; 2 Nanjing Jingwei Mining Technology Research Institute Company Limited, Nanjing, 210003 China; 3 Guizhou Institute of Technology, Guiyang, 550025 China

**Keywords:** Deep soft rock roadway, Floor heave mechanism, Control technique, Numerical simulation, Banji Coal Mine, Engineering, Natural hazards, Solid Earth sciences

## Abstract

Aiming at the severe floor heave occurring at the machine head section of the belt conveyor downhill roadway in the 1105 mining area of Banji Coal Mine, Bozhou, Anhui, a systematic investigation was conducted using mechanism analysis, numerical simulation, and on-site industrial testing to elucidate the multifactorial mechanism and control technique of floor heave in deep soft rock roadways. The dominant factors contributing to roadway floor heave were identified as high horizontal tectonic stress, weak floor lithology, high water content in the floor strata, and deficiencies in the original support system. Subsequently, the loosening zone of the roadway floor was detected, and the development characteristics of floor plastic deformation and deformation concentration were analyzed in detail. On this basis, the reasonable effective depth for grouting reinforcement was determined. Accordingly, an integrated floor heave control technique combining deep-hole high-pressure grouting and drainage was proposed to reinforce and strengthen the roadway structure. A numerical model was then established to comparatively analyze roadway deformation over a three-month period under conditions with and without reinforcement. The results indicated that the overall stress state of the surrounding rock was significantly improved after reinforcement, leading to effective control of floor heave deformation, while deformation of the roof and sidewalls was simultaneously mitigated. Furthermore, on-site monitoring results demonstrated that 30 d after grouting, floor subsidence, inwardconvergence of the two sides, and floor heave of the roadway all reduced quickly, and the deformation gradually stabilized. These findings confirm that the proposed floor heave control technique can effectively suppress deformation of the roadway surrounding rock and ensure safe and stable operation.

## Introduction

Floor heave in coal mine roadways is one of the most common surrounding rock deformation disasters during deep mining and poses a serious threat to mine safety and long-term roadway stability. With increasing mining depth, the coupled effects of high in-situ stress, weak rock strata, groundwater action, and mining disturbance significantly intensify floor heave, making it a critical technical challenge that restricts efficient coal mine production^1–4^. Statistics indicate that more than two-thirds of deep coal mine roadways in China experience floor heave to varying degrees, and in some mines the heave rate exceeds 500 mm per month. Such severe deformation results in roadway cross-section shrinkage, transportation obstruction, and may further induce secondary disasters, including roof fall and sidewall instability, thereby seriously threatening safe and efficient mine operation^5–8^. Therefore, an in-depth investigation of the multifactorial mechanism of floor heave in deep soft rock roadways and the development of targeted control technologies are of great significance for safe coal mining.

Extensive studies have been conducted worldwide on the multifactorial mechanisms of floor heave in deep soft rock roadways^9–14^. Gangye Gu^15^ demonstrated that floor heave in deep coal mine roadways is not solely governed by weak floor strata but is also strongly influenced by the coupled pressure relief of the roof and sidewalls. Ivan Sakhno^16^ reported that soft rock undergoes softening and swelling after water absorption, leading to the initial formation of a vertical expansion and plastic failure concentration zone within the roadway floor, which subsequently propagates outward and drives the evolution of floor heave. Zexin Li^17^ proposed that floor heave represents a process of overall instability of the surrounding rock system, in which lateral constraint weakening, sidewall failure, and floor plastic zone expansion interact and evolve synergistically, forming a coupled instability pattern characterized by “sidewall failure weakening floor bearing capacity and floor heave further aggravating sidewall convergence”. Yuping Fu^18^ indicated that the roadway floor experiences an evolutionary process of “structural relaxation—hardening loading—fracture instability”, during which stress is repeatedly superimposed and transmitted to deeper strata, resulting in cumulative floor damage, plastic zone expansion, and ultimately intense floor heave. Weijun Wang^19^ attributed floor heave in roadway along goafs to non-uniform deformation of the floor rock mass under the combined effects of high stress from solid coal ribs and stress release from the goaf, causing plastic flow and shear failure of the floor strata toward both the roadway and the goaf. Quansheng Liu^20^ suggested that mining disturbance induces stress redistribution in the floor, leading to combined tensile and shear deformation, while the loose surrounding rock structure and insufficient floor bearing capacity promote stress concentration at floor corners and inward expansion, eventually triggering floor heave failure.

Meanwhile, substantial progress has been made in the development of floor heave control techniques for coal mine roadways. Lining Feng^21^ derived a calculation formula for floor failure depth by establishing a roadway floor slip model and proposed an anti-slip floor heave control technique in combination with a steel pipe concrete pile structure. Chuang Sun^22^ identified deficiencies in the original support scheme through numerical simulation and proposed an optimized support system involving the installation of floor bolts, extension of bolt length, and application of preload. Furong Tang^23^ revealed the fracture evolution characteristics of deep soft rock roadways based on field tests and numerical simulations and identified an “optimal grouting timing” interval bounded by two acceleration inflection points, which was subsequently validated through on-site application. Donghuang Shang^24^ achieved coordinated enhancement of multidirectional load-bearing capacity of the surrounding rock by reinforcing the roof and sidewalls with layered anchor cables to form a stable ring, excavating pressure relief grooves in the floor, and combining grouting and gangue filling with floor corner anchor bolts. Guangyuan Yu^25^ developed a floor heave control technique for roadways along goafs based on roof cutting and pressure relief, in which directional pre-split blasting was employed to interrupt roof stress transfer, optimize the surrounding rock stress environment, reduce support pressure on solid coal ribs, and thereby decrease the load transmitted to the floor. Hongyang Liu^26^ proposed a collaborative control technique aimed at strengthening the full-section load-bearing structure of surrounding rock through combined application of backfilling behind U-shaped steel supports, shallow and deep floor grouting, and inverted arch installation.

Despite these advances, the factors inducing roadway floor heave are numerous, and the corresponding disaster mechanisms remain complex and diverse, indicating that current understanding is still insufficient. Researches on floor heave control technologies in roadways can be currently characterized by the coexistence of multiple theories and technologies due to significant differences in geological conditions across various mining areas and the diversity of in-situ stress conditions, with various theories and technologies still requiring further verification and optimization. Aiming at the severe floor heave occurring at the machine head section of the belt conveyor downhill roadway in the 1105 mining area of Banji Coal Mine, the causes and mechanisms of floor heave were specifically analyzed in this paper. Based on this and the determination of floor loose zone range, an integrated floor heave control technique combining deep-hole high-pressure grouting and drainage was proposed and implemented, and its effectiveness was verified through numerical simulation and on-site industrial testing to ensure stable and normal roadway operation. The findings can provide a theoretical foundation and technical reference for floor heave treatment in roadways under similar geological conditions.

## Engineering overview

### Geological overview

**Fig. 1:**
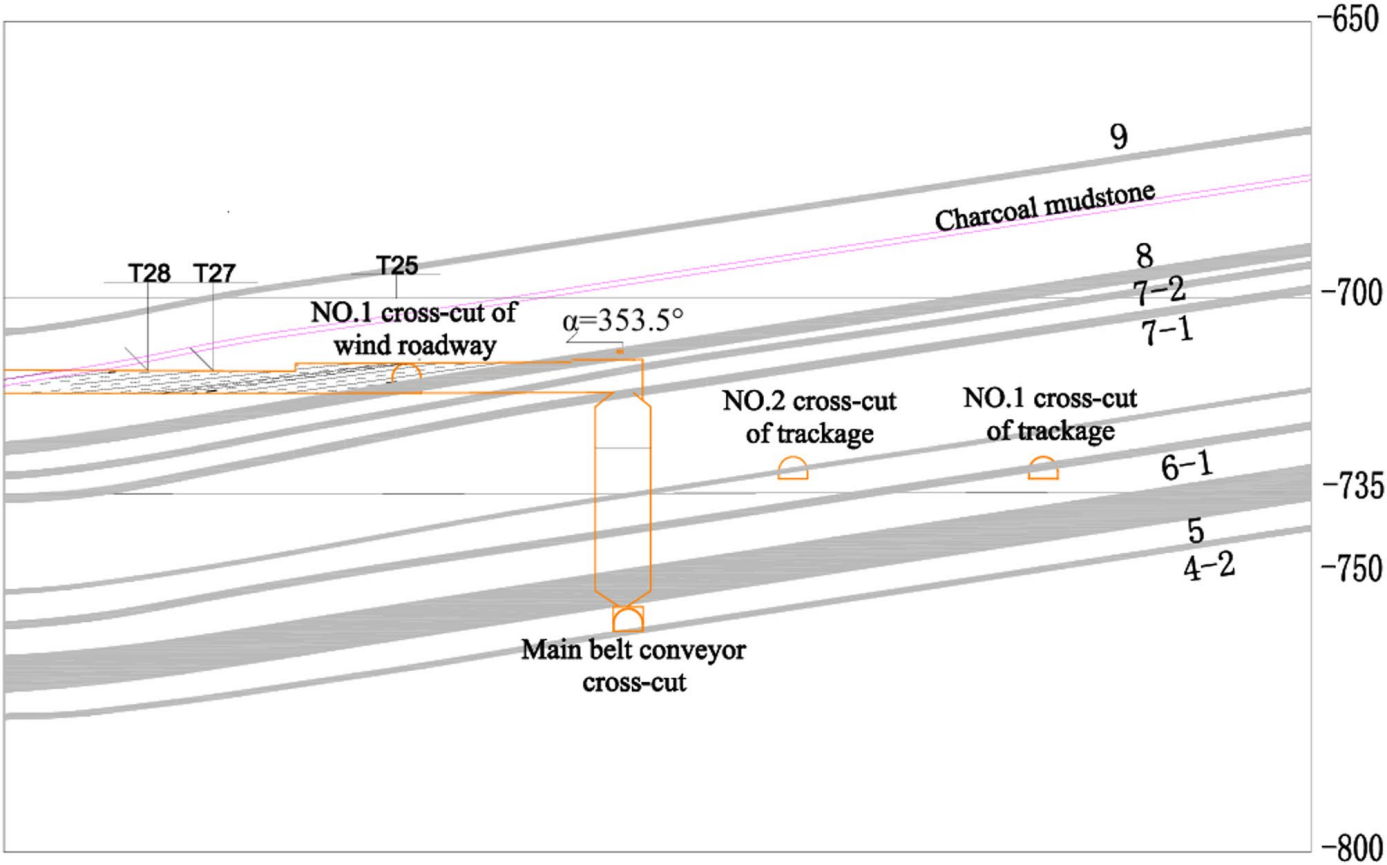
The profile of the machine head section of belt conveyor downhill roadway.

Banji Coal Mine is located in Huji Town, Lixin County, Bozhou City, Anhui Province. The machine head section of the belt conveyor downhill roadway in the 1105 mining area serves as a crucial passage within the mine’s transportation system. Important underground engineering structures, including gangue bins, coal bins, main transportation belt stone gates, and return air stone gates, are distributed around this section. The stability of this roadway directly impacts the safe and efficient production of the entire mining area. Its cross-section is shown in Fig. 1.

The roadway is buried at a depth of -740 m and has a total length of 40 m. Its cross-section adopts a rectangular wall and semi-circular arch structure, with a clear width of 5.5 m, a wall height of 2.45 m, and a cross-sectional area of approximately 28.6 m². The geological conditions of the surrounding rock are as follows: the immediate roof consists of fine sandstone, while the main roof is sandy shale; the immediate floor is shale, and the main floor is sandy shale. The lithology of the roadway roof and floor strata is listed in Table 1.

**Table 1:** The lithology of roadway roof and floor strata.

Order Number	Lithology	Thickness /m	Lithological description
1	Sandy mudstone	12.7	Dark grey, massive, with more sand in the upper part and less in the lower part.
2	No.7 coal seam	1.2	Black massive, locally powdery, mainly dark coal.
3	Mudstone	4.1	Dark grey, dense massive, with numerous plant root fossils.
4	Sandy mudstone	3.1	Grey, massive, with more sand in the middle, with plant root fossils.
5	Fine sandstone	8.9	Light grey, with quartz, interbedded with black muddy bands.
6	Mudstone	8.7	Dark grey, dense, massive, with numerous plant root fossils.
7	Sandy mudstone	4.6	Dark grey, massive, with more sand in the upper part and less in the lower part.
8	**No.6 coal seam** **(where the roadwayl is located)**	1.2	Black granular and powdery, composed of dark coal and bright coal.
9	Mudstone	9.0	Dark grey to black, dense, massive, partially carbonized.
10	Fine sandstone	6.5	Light grey, mainly with a silt structure, locally interbedded with thin muddy layers.

### Original support scheme

**Fig. 2:**
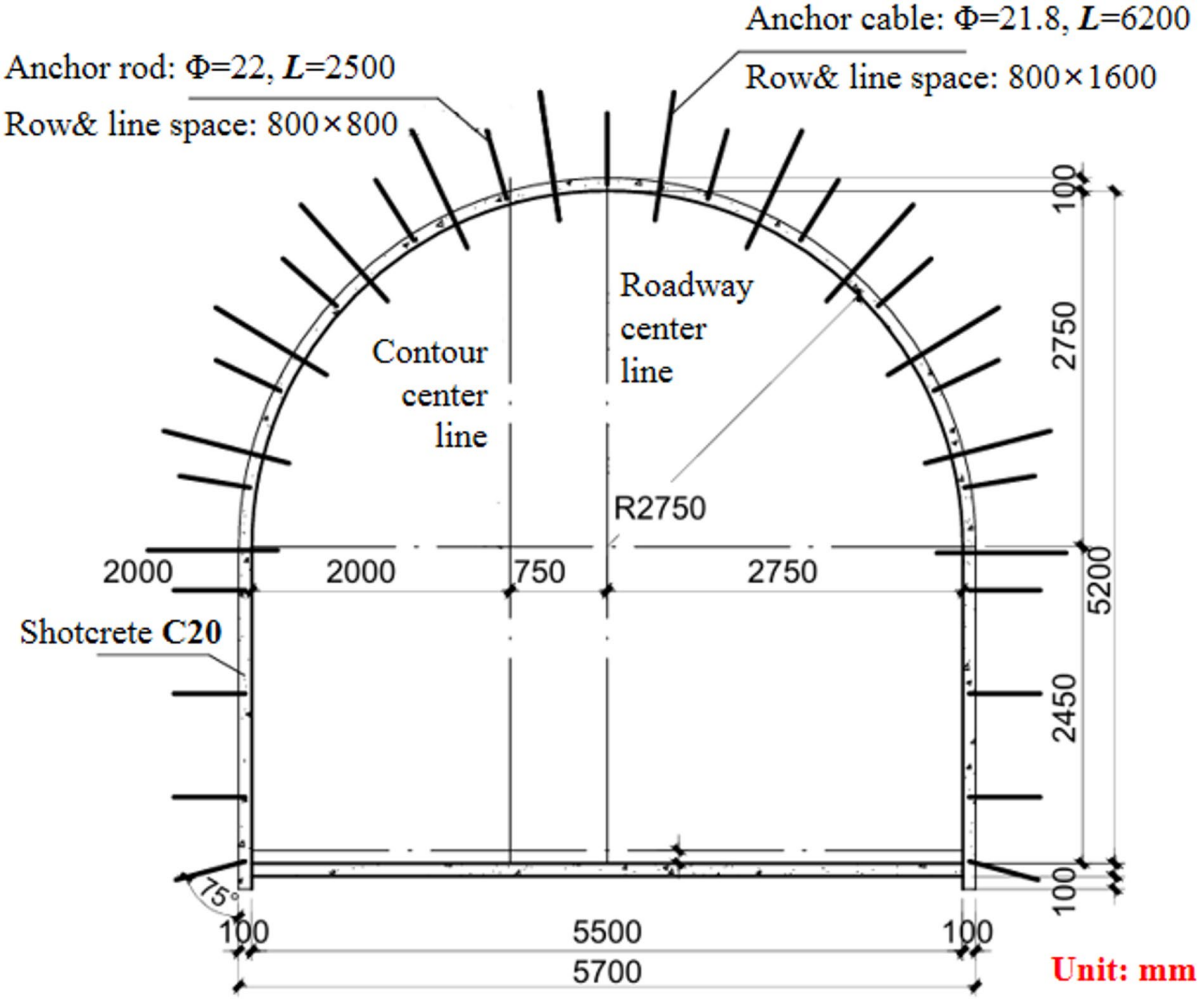
Original support design of the roadway.

The original support scheme of the machine head section roadway is shown in Fig. 2. A combined support system consisting of bolts, mesh, and cables was adopted.

In the roadway arch, left-handed ribless threaded steel anchor rods with a diameter of 22 mm and a length of 2500 mm were installed, while solid anchor cables with a diameter of 21.8 mm and a length of 6200 mm were employed. The spacing of anchor rods was set at 800 mm × 800 mm, and the anchor cables were arranged at a spacing of 800 mm × 1600 mm. The sidewalls were also reinforced using left-handed ribless threaded steel anchor rods with a diameter of 22 mm and a length of 2500 mm, arranged at a spacing of 800 mm × 800 mm. The support system was integrated with a welded metal mesh fabricated from smooth steel bars with a diameter of 6 mm. After completion of the support installation, a 100 mm thick layer of C20 concrete was sprayed to seal the surface, thereby enhancing the integrity and load-bearing capacity of the surrounding rock.

## Damage characteristics and mechanisms of roadway floor heave

### Damage characteristics of floor heave

**Fig. 3:**
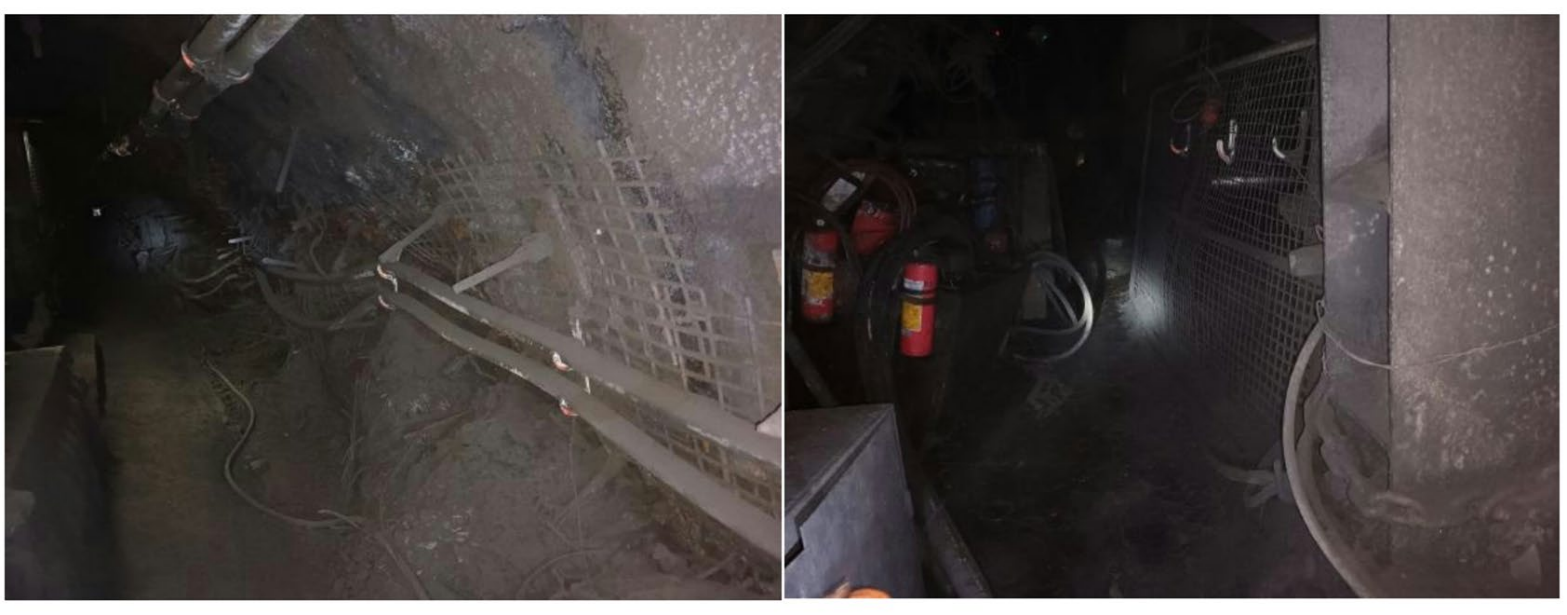
Deformation of the head section roadway.

Based on on-site investigations and deformation monitoring, the primary manifestations of surrounding rock deformation and failure in the machine head section roadway were floor heave and rib extrusion, as shown in Fig. 3.

The characteristics of roadway deformation and failure can be summarized as follows.

(1) The damaged area of the surrounding rock was concentrated in the middle of the floor and the lower half of the two ribs, with the floor heave rate reaching up to 150 mm/month. The floor exhibited bulging, crack propagation, and localized rock fragmentation, with mud seepage observed in some cracks. The large deformation of the surrounding rock leaded to significant tilting of mechanical and electrical equipment, severely affecting the normal operation of the roadway.

(2) The continuous deformation of the surrounding rock resulted in the formation of intersecting cracks on the surface of the sprayed concrete support. In some areas, the sprayed layer peeled off, losing its original functions of enclosure and load-bearing support. Additionally, due to the incoordination of deformation, slippage occurred between the support structures and surrounding rock, leading to widespread anchor failure and the formation of voids.

(3) Despite multiple repairs, the surrounding rock has not stabilized, and deformation and damage continue to evolve in the original damaged zone and its surrounding areas. Cracks repeatedly develop, and new cracks propagate along weak structural zones in the surrounding rock, creating a vicious cycle of “repair-damage -second repair - second- damage…”. During this cycle, rock spalling and fragmentation intensify.

Frequent maintenance operations not only increase production costs but also severely restrict the safe and efficient operation of the mine. Therefore, it is urgent to optimize the original support system and implement more effective floor and rib control technologies to enhance the overall stability of the surrounding rock, ensuring the long-term safety and normal operation of the roadway.

### Damage mechanism analysis of floor heave

Floor heave in the machine head section roadway is essentially the result of squeezing and softening of the floor strata induced by the lateral deformation and expansion of the surrounding rock on both sides under high stress conditions. The contributing factors can be summarized as follows.

**Fig. 4:**
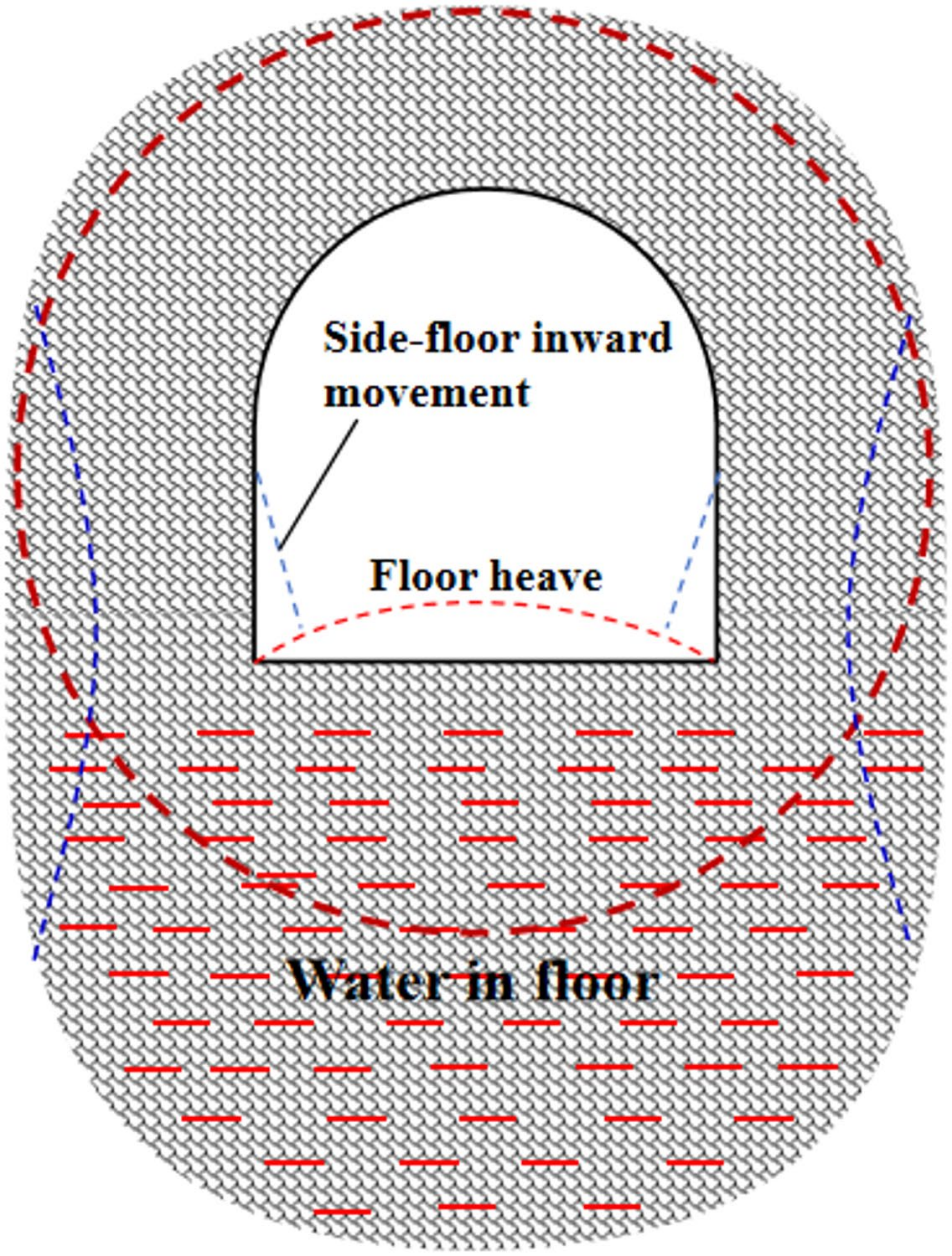
Mechanism for extrusion-type floor heave formation.

(1) High ground stress. The roadway is buried at a depth of approximately − 740 m, where it is subjected to a deep-seated high-stress environment. The substantial vertical ground stress associated with the large burial depth induces pronounced inward deformation of the roadway ribs and floor. This deformation continuously compresses the floor loosening zone, forcing it to deform upward and ultimately resulting in floor heave.

(2) Weak floor lithology. The roadway floor is predominantly composed of shale, which is characterized by low strength and a strong tendency toward mudification upon contact with water. Meanwhile, a considerable amount of water is accumulated within the floor strata, leading to an overall low load-bearing capacity. Under prolonged groundwater soaking, shale undergoes mudification and erosion. Mudification increases shale porosity and enhances groundwater permeability, thereby causing a marked deterioration in its mechanical properties. Erosion further disrupts the continuity and integrity of the rock mass structure, weakening its resistance to ground stress. As a consequence, the floor strata are unable to withstand the compressive squeezing exerted by both sides of the roadway, resulting in severe compressive deformation.

(3) Inadequate original support. Roadway support plays a critical role in controlling surrounding rock deformation and maintaining structural stability. A rational support system can effectively restrain deformation development, delay damage evolution, and inhibit crack propagation in the surrounding rock. Compared with the roof and ribs, the floor tends to develop a larger loosening zone in the absence of effective support. This zone readily acts as a pathway for groundwater migration or a water-bearing region, leading to significant softening of the floor rock mass. The softened rock mass exhibits a substantially reduced resistance to compressive deformation, and its deformation is therefore preferentially directed toward the free space at the bottom of the roadway. Simultaneously, insufficient high-strength support on both sides of the roadway allows lateral displacement of the ribs under high vertical ground stress, which further compresses the floor loosening zone and ultimately induces floor heave. The coordinated deformation characteristics and underlying mechanisms of the surrounding rock are illustrated in Fig. 4.

(4) The collaborative control system has not been established. Due to inadequate support and a high degree of fragmentation, the surrounding rock in floor has become a weak link in roadway deformation, can not form an effective collaborative control system structure with the ribs and roof, significantly weakening the overall capacity of roadway surrounding rock to resist deformation.

## Reinforcement support scheme design

### Detection of Floor Loosened Zone and Determination of Reinforcement Depth

Previous studies have demonstrated that roadway floor heave is strongly governed by the development extent of the deep-seated loosening zone within the floor strata. The magnitude of floor heave deformation is closely associated with whether the loosening zone is effectively reinforced, rather than being controlled solely by localized shallow damage. Consequently, accurate identification of the floor loosening zone and determination of the corresponding grouting reinforcement depth are critical for effective floor heave control. A bracket-type ZYJ-750/200 hydraulic rotary drilling rig was employed to drill vertical exploration boreholes in the floor of the machine head section roadway for loosening zone investigation. The criteria for determining the extent of the floor loosening zone before and after floor grouting are described as follows.

(1) Before grouting. The extent of the floor loosening zone can be identified based on variations in drilling speed, water discharge characteristics from the borehole, and the condition of drill cuttings. When drilling speed decrease markedly and the cuttings exhibits mudification accompanied by continuous water discharge, the rock mass can be judged to be in a loose and damaged state. When drilling speed increase significantly and the integrity of the rock mass is evidently improved, this position can be identified as the boundary of the floor looseing zone.

(2) After grouting. The presence of cementitious components in the drill cuttings can be adopted as the primary indicator for determining the effective range of grouting reinforcement.

**Fig. 5:**
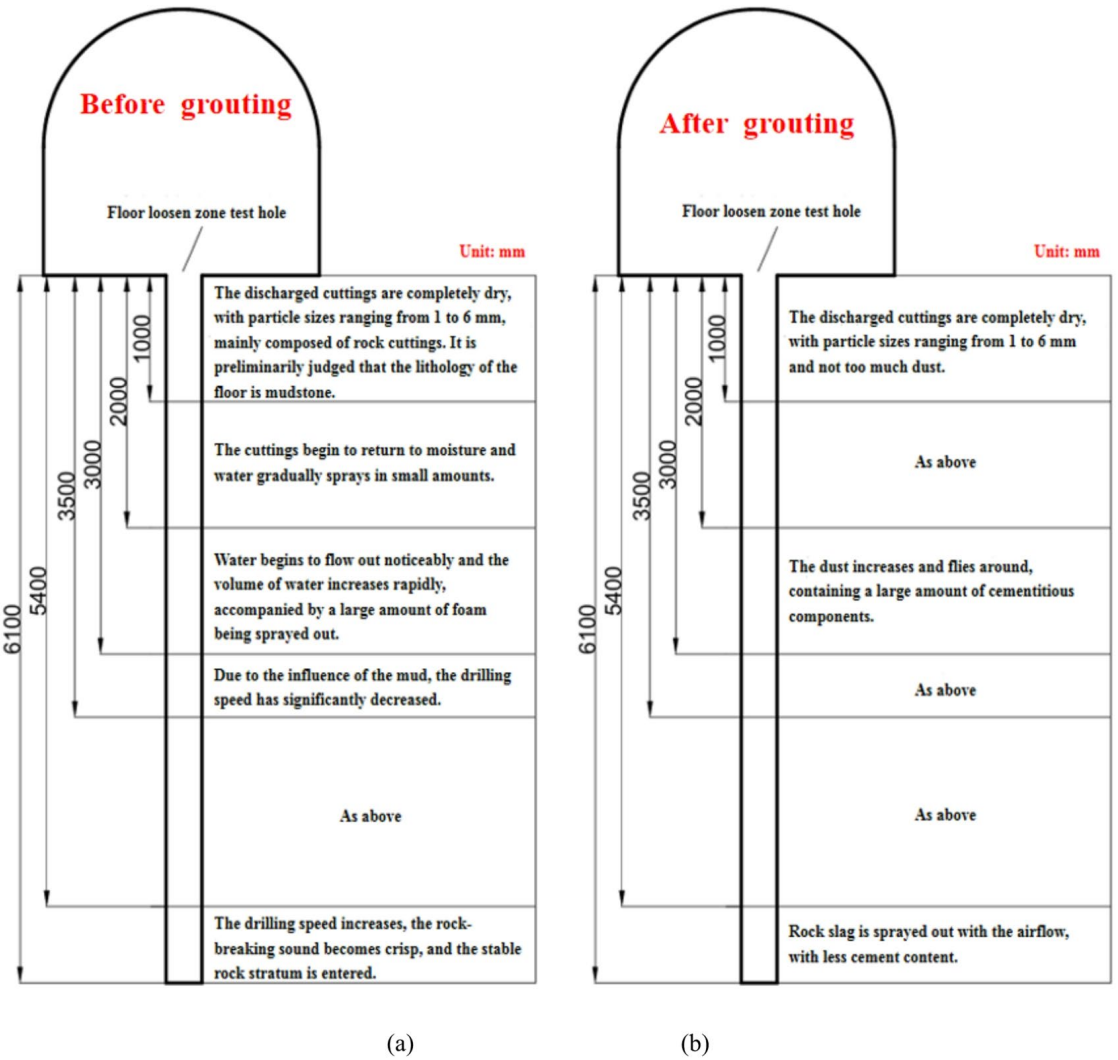
Detection results of floor loosen zone by drilling.

The extent of the floor loosening zone was investigated on-site both before and after floor grouting. The borehole peeping results are shown in Fig. 5.

(1) The drilling exploration results obtained before grouting indicate that. As shown in Fig. 5a, the rock mass within a depth range of 0–5400 mm in the roadway floor is highly fragmented and loose, with well-developed fractures and continuous water discharge. This interval can therefore be identified as the floor loosening zone. When the drilling depth exceeded 5400 mm, drilling speed increased markedly and the integrity of the rock mass improved significantly. Accordingly, 5400 mm was determined as the boundary of the floor loosening zone.

(2) Drilling exploration was conducted again 15 d after floor grouting. As shown in Fig. 5b, cementitious components were detected in the drill cuttings within the depth range of 0–5400 mm, whereas no cement components were observed in the cuttings below 5400 mm. This result indicates that the grout predominantly diffused within the floor loosening zone and achieved effective filling and bonding. The reinforcement range is therefore generally consistent with the development extent of the floor loosening zone.

Based on the detection results of the floor loosening zone, the grout diffusion characteristics, and the engineering safety factor, the grouting reinforcement depth for the floor in the machine head section roadway can be determined to fully cover the 5400 mm loosening zone as the control range, and the vertical depth of the grouting boreholes can be designed as 8000 mm (The method for setting the vertical depth of the grouting boreholes is as follows: based on the grouting scheme parameters and design experience, the grouting diffusion radius can be determined as 0.6 times the grouting pipe spacing, i.e., 1200 mm. Then, with a safety margin of *f* = 1.2, the vertical depth of the grouting boreholes can be calculated as (5400 + 1200)×1.2 ≈ 8000 mm).

### Principle analysis of reinforcement support design

(1) Current status and problems of the roadway.

**Fig. 6:**
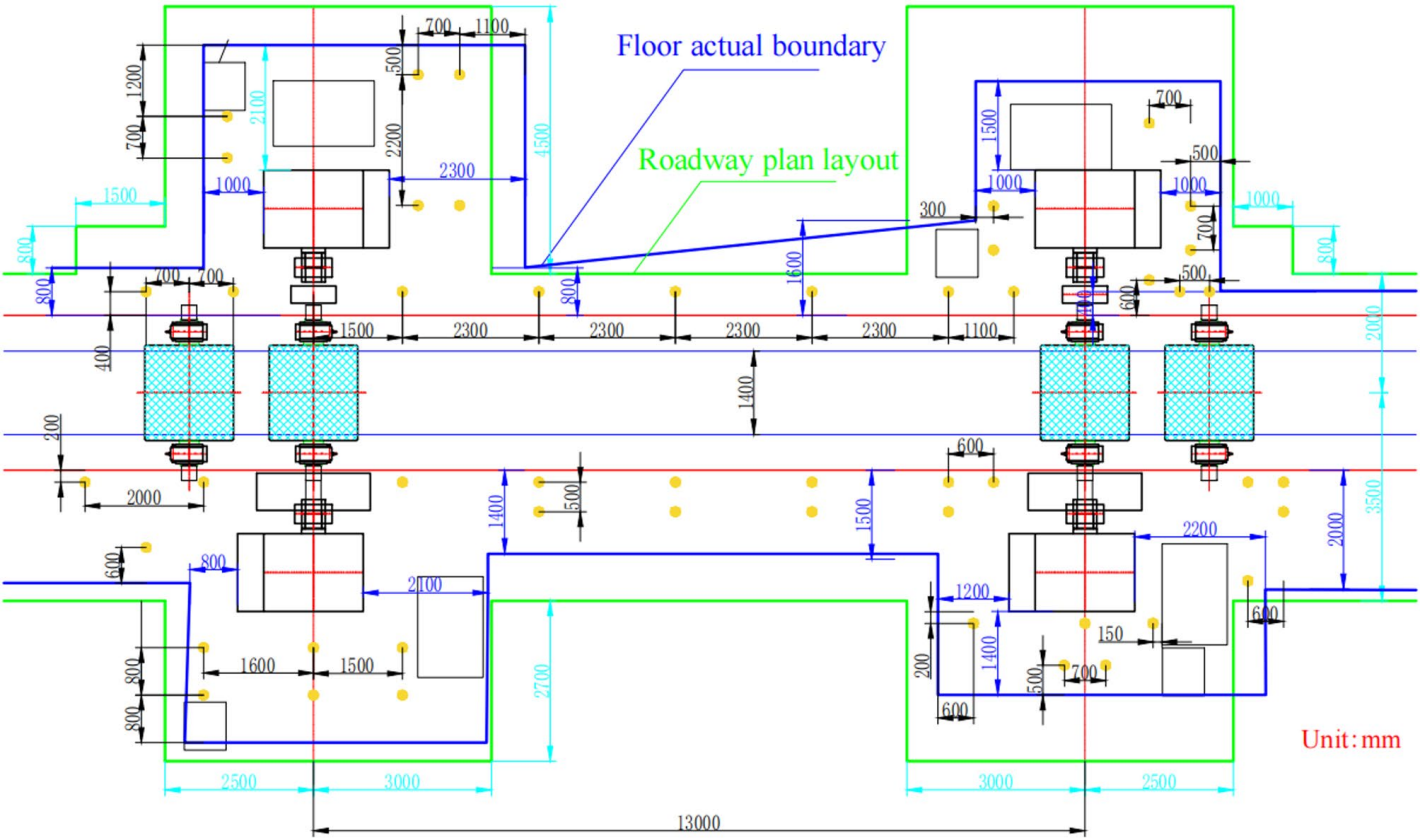
Plan view of the face section tunnel and grouting hole layout.

Significant deformation has occurred in the floor and ribs of the target roadway in the 1105 mining area, and the roadway cross-section remains unstable despite multiple repair measures. The roadway layout and the actual boundary of the floor are shown in Fig. 6, which indicates a wide development range of the floor loosening zone and pronounced convergence of the two ribs.

On-site investigations reveal that the roadway floor in this section is mainly composed of shale with a loose structure and poor integrity, making it susceptible to creep softening and shear failure under the combined effects of high ground stress and groundwater. Local weak zones are developed in the ribs, whereas the roof rock mass remains relatively intact and exhibits limited deformation. Owing to the absence of systematic floor support, the floor has become the primary zone of concentrated deformation in the surrounding rock.

The above analysis demonstrates that the original support system provides insufficient confinement and fails to satisfy the requirements for long-term stability of deep weak roadway. Accordingly, a deep-hole high-pressure grouting and drainage integrated support scheme was proposed to reinforce the roadway floor.

(2) Mechanism of reinforcement support scheme.

The deep-hole high-pressure grouting and draining integrated support scheme improves the stress and deformation state of the floor through the synergistic action of “grouting reinforcement and liquid discharge with pressure relief”. During the grouting stage, high-pressure grout propagates along fractures and within the floor loosening zone, effectively filling pores and cementing fragmented particles. This process markedly enhances the cohesion, integrity, and elastic modulus of the floor rock mass, forming a continuous and dense reinforced body within the weak and shear-prone zones. During the liquid discharge stage, pore water pressure and localized fluid-induced stress within the loosening zone are directionally released, thereby weakening creep softening under seepage conditions and reducing stress concentration, which suppresses the initiation and development of shear slip. Meanwhile, the strengthened floor surrounding rock can be organically integrated with the support structures of the roadway ribs and roof, constructing an integrated surrounding rock bearing system, which can greatly enhance the overall capacity of roadway surrounding rock to resist deformation.

Under the combined effects of grouting, liquid discharge, and the integrated surrounding rock bearing structure, the stress distribution of the roadway floor evolves from a highly unbalanced state toward a relatively uniform state. The extent of the plastic zone is significantly reduced, the tendency for deformation concentration is effectively alleviated, and the mechanical behavior of the surrounding rock gradually transforms from softening deformation to stable load-bearing. This provides a fundamental support condition for the long-term stability of deep roadways with weak floor strata.

### Deep-hole high-pressure grouting and draining integrated design

(1) Layout of grouting holes.

The layout of the floor grouting holes is shown in Fig. 6, where the yellow markers indicate the locations of the grouting hole openings on the floor surface. To improve the spatial coverage of the floor loosening zone, the grouting holes were arranged in a multidirectional combination. The angle between the hole axis and the horizontal plane was uniformly set at 70°. A total of 55 grouting holes were arranged along the roadway strike, perpendicular to the strike, and at an angle of 45° to the strike, so as to accommodate the non-uniform deformation characteristics of the roadway floor.

(2) Specifications and structure of grouting holes.

**Fig. 7:**
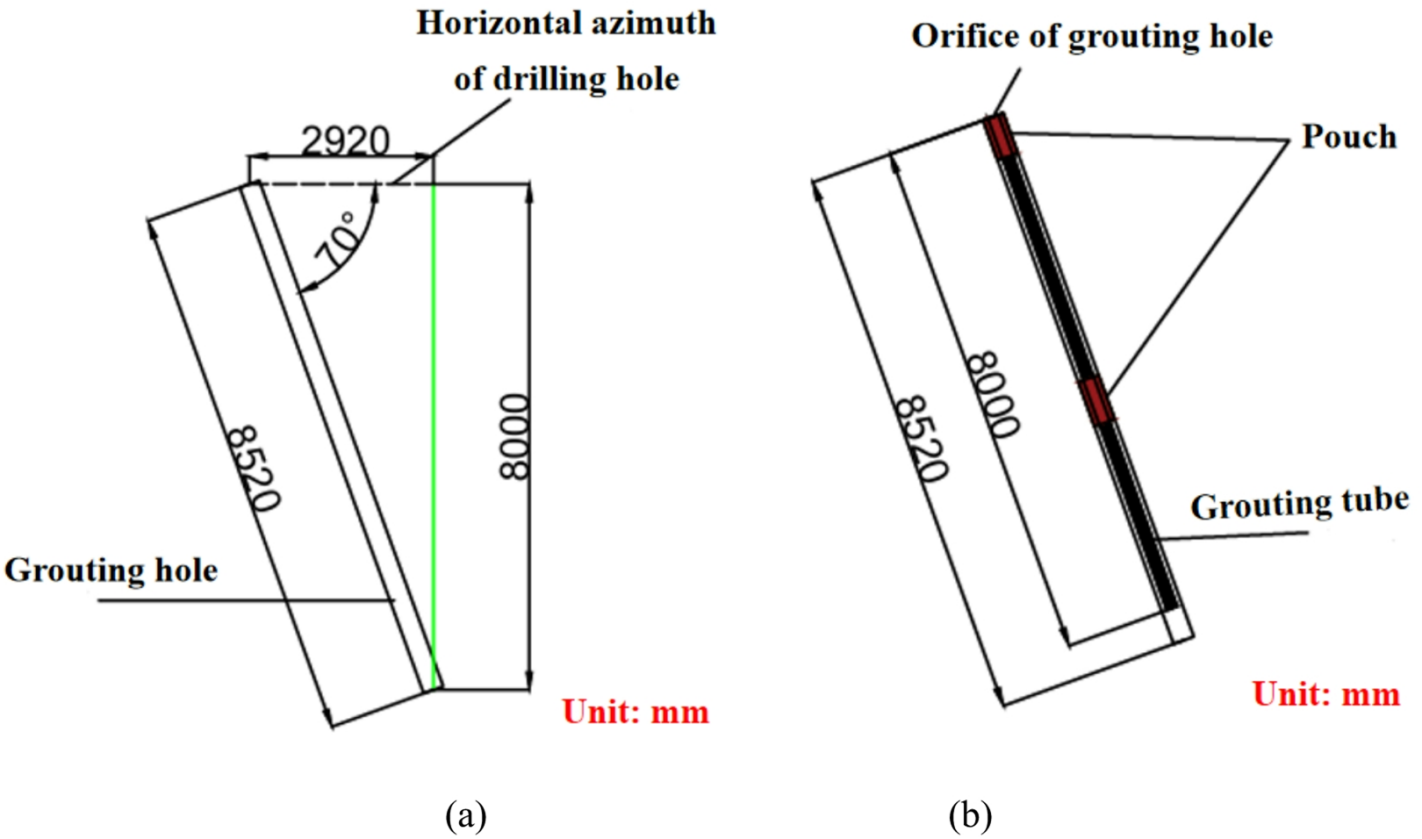
Drill hole profile. (**a**) Horizontal projection of borehole, (**b**) Grouting pipe structure.

The structural parameters of all grouting holse were kept consistent. Each hole had a vertical depth of 8.0 m, an inclined depth of 8.52 m, and a diameter of 65 mm. A steel grouting pipe was installed inside each hole, with evenly distributed perforations along the pipe wall to ensure effective grout diffusion within the floor loosening zone. The detailed structural parameters are shown in Fig. 7.

(3) Hole-sealing and grouting process.

**Fig. 8:**
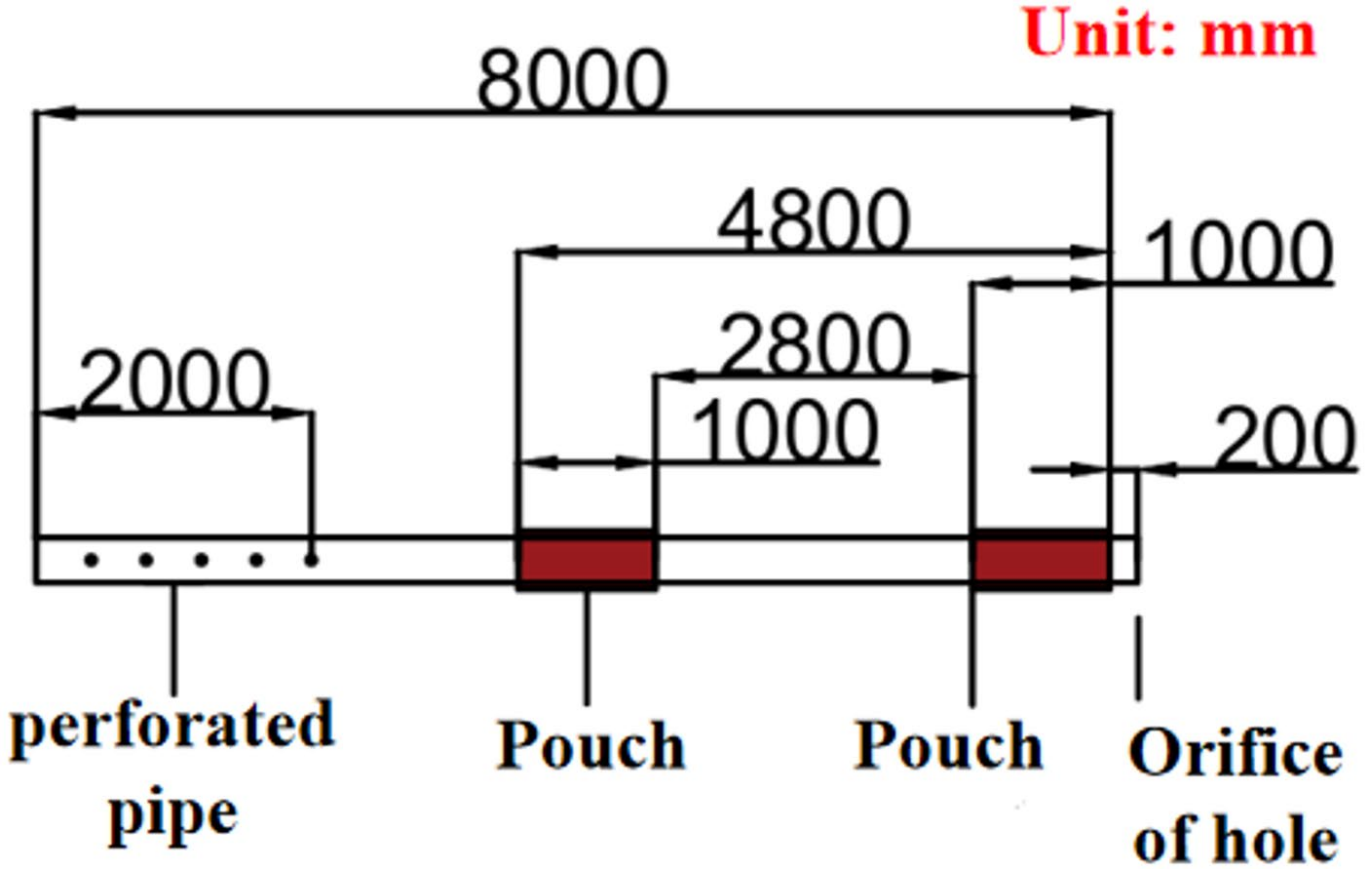
Schematic diagram of grouting sealing structure.

The hole-sealing process adopted the “double-plug-and-grout” technique, with a sealing length of 4.8 m, as shown in Fig. 8. After completion of hole sealing, grouting was carried out sequentially for each borehole. The grouting pressure was controlled within a range of 0 ~ 20 MPa. The average grout consumption per hole was approximately 1600 kg, calculated based on the mass of dry powder. Grouting was terminated when the grouting pressure reached the upper limit and the grout intake decreased significantly.

(4) Grouting materials and slurry preparation.

The grouting material consisted of JSZK-80 high-performance inorganic environmentally friendly filling and reinforcing material for mines mixed with 45% tap water. During slurry preparation, clear water was added to the mixing drum according to the designed water-cement ratio. After the mixer was started, the dry powder was slowly introduced and continuously stirred for 6 ~ 8 min to obtain a homogeneous slurry. The prepared slurry exhibited good fluidity and early-strength performance, satisfying the requirements of diffusion and load-bearing capacity in deep-hole high-pressure grouting and drainage integrated reinforcement.

(5) Grouting quality detecting.

Use a column-mounted floor cable bolting rig to construct a 5 m deep borehole with dry drilling. If the ejected material consists entirely of dust scattering in all directions but the drilling speed is relatively fast, the grouting quality is basically qualified. If scattered dry powder is ejected and the drilling speed is slow, the grouting quality is good. If wet rock debris is ejected from the floor during the drilling process, the grouting quality is unqualified. If water is still ejected during the drilling process, the grouting quality is seriously unqualified.

## Numerical simulation and results analysis

Based on the geological and engineering conditions of the machine head section of the belt conveyor downhill roadway in the 1105 mining area of Banji Coal Mine, numerical calculation models of the roadway with and without reinforcement support were established using FLAC3D software. Comparative analyses of the stress distribution and deformation characteristics of the surrounding rock were conducted under the two conditions, with the aim of quantitatively evaluating the effectiveness of the proposed reinforcement support scheme.

### Numerical modeling

(1) Model overview.

**Fig. 9:**
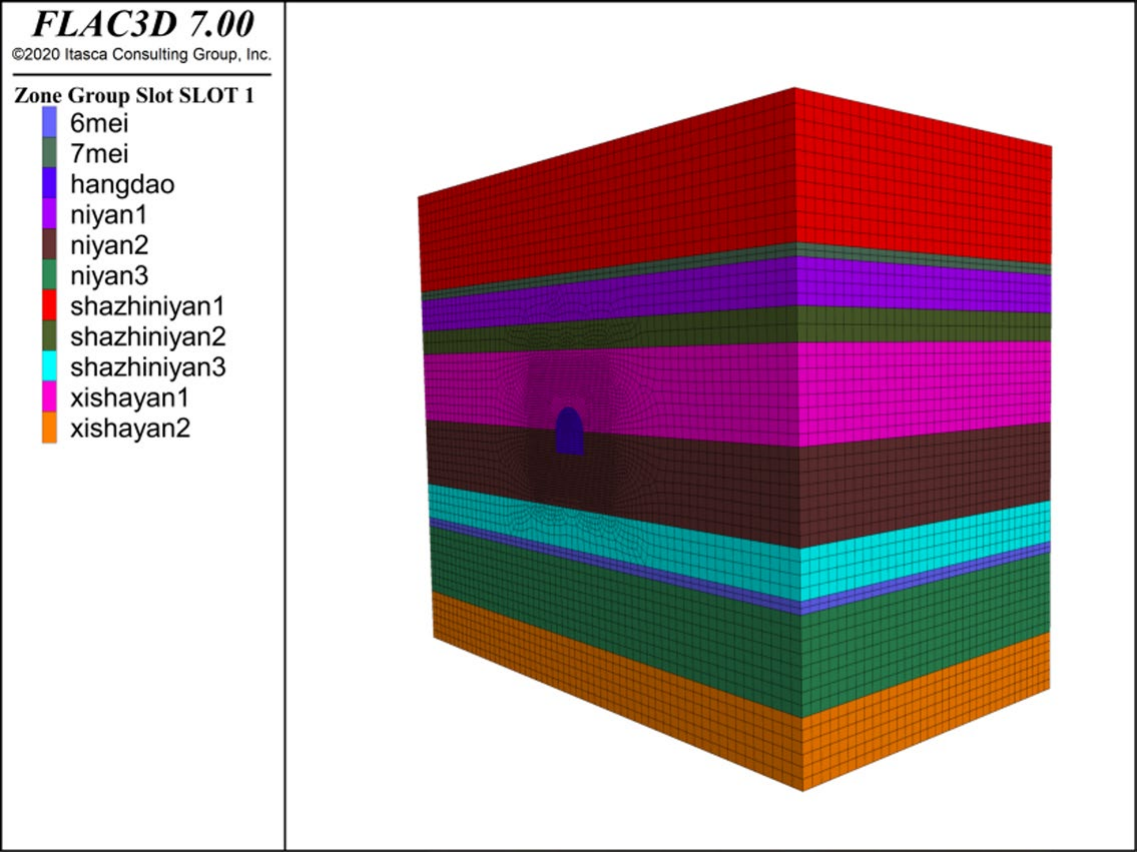
Numerical model.

The roadway numerical model was established using FLAC3D software, as illustrated in Fig. 9. The model comprises 10 rock strata and has dimensions of 70 m in the transverse direction (X-axis), 40 m in the longitudinal direction (Y-axis), and 60 m in the vertical direction (Z-axis), with the roadway located at the center of the model. To achieve a balance between computational accuracy and storage efficiency, mesh refinement was applied in the vicinity of the roadway. The overall mesh layout exhibits a radially distributed pattern, characterized by dense elements near the roadway and progressively coarser elements outward. In total, the model contains 662,546 hexahedral elements and 519,280 grid nodes.

**Fig. 10:**
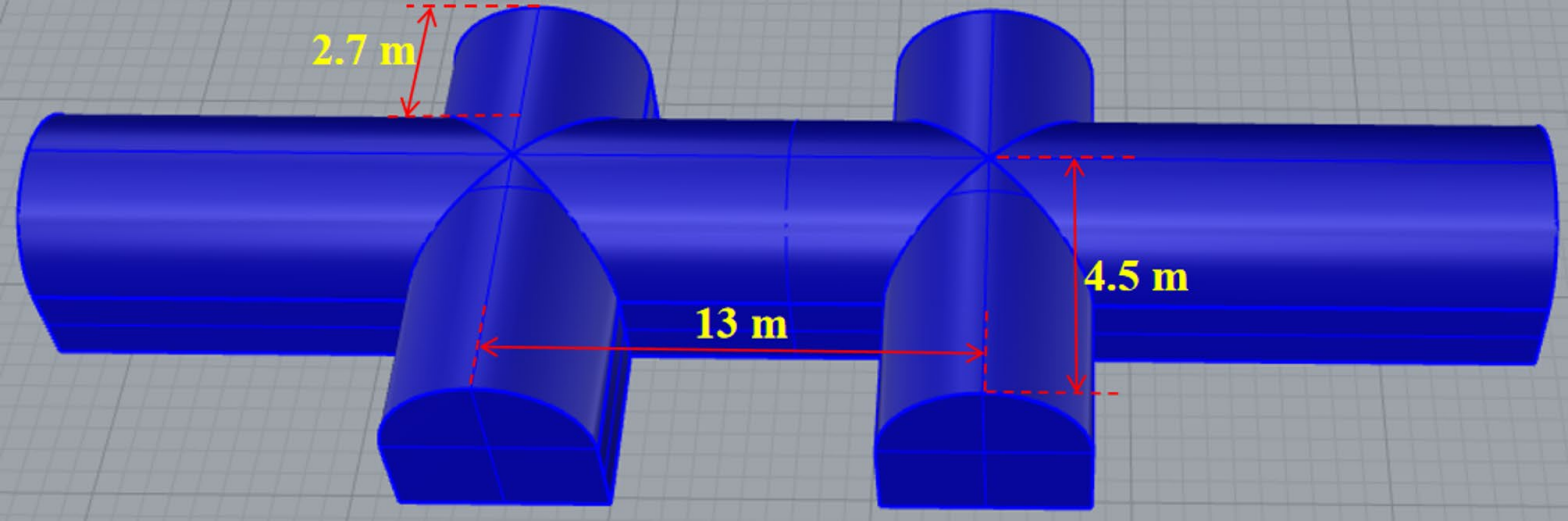
Three-dimensional view of the head section.

Chambers were arranged on both sides of the machine head section roadway, with the same cross-section form as the main roadway. The left chambers have a strike distance of 2.7 m, and the right ones have a strike distance of 4.5 m, both connected to the machine head section roadway, as shown in Fig. 10. This spatial arrangement forms a “main roadway-side chamber” combined structure in the calculation model, whose overall stress characteristics differ from those of conventional single roadways, exerting certain influence on surrounding rock stability and floor heave control.

(2) Boundary conditions and material parameters.

The boundary conditions of the model set as follows: a uniform vertical load of 18.5 MPa was applied at the top of the model to simulate the weight of the overlying strata, based on the roadway burial depth. The lateral pressure coefficient was set to 1.3 according to in-situ ground stress testing results. Fixed displacement constraints were applied to the sides, front and back, and bottom of the model.

The mechanical behavior of the surrounding rock during excavation and initial support was modeled using the Mohr-Coulomb constitutive model. The physical and mechanical parameters of the rock were obtained through laboratory testing, and the specific parameter settings are listed in Table 2.

**Table 2:** Mechanical properties of coal and rock.

Rock type	Weight (kN /m^3)^	Elastic modulus(GPa)	Shear modulus(GPa)	Internal friction angle(degree)	Cohesion (MPa)	Poisson’s ratio
Sandy mudstone	2550	4.45	1.72	31	4.6	0.29
No.6 coal seam	1400	1.49	0.56	20	1.2	0.32
Mudstone	2461	1.13	0.42	28	0.9	0.33
Fine sandstone	2597	8.48	3.37	36	10.1	0.26

**Fig. 11:**
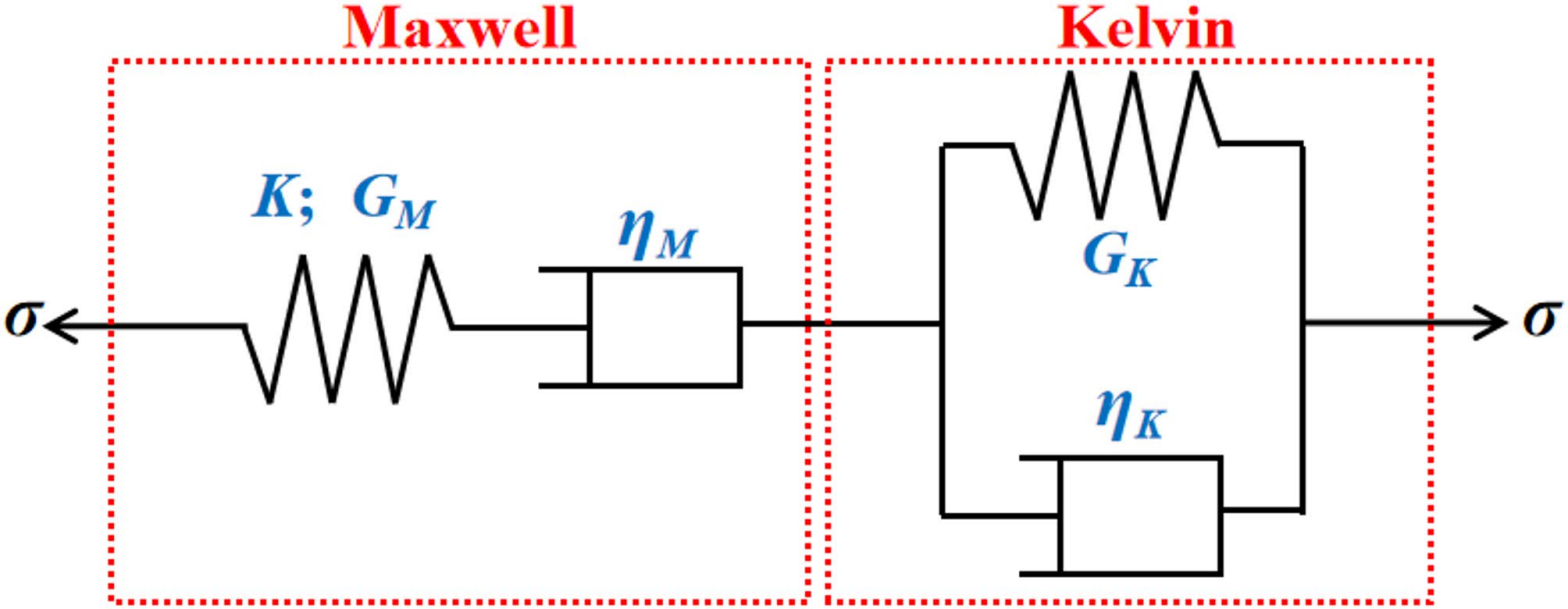
Burgers constitutive model.

To simulate the time-dependent deformation behavior of the soft rock surrounding rock under long-term loading conditions, the Burgers constitutive model was introduced after roadway excavation to characterize creep deformation. The Burgers model is composed of a Maxwell element and a Kelvin element connected in series, as shown in Fig. 11. In the figure, *K* is the bulk modulus of rock mass, *σ* is the load applied to rock mass, *G*_M_ and *G*_K_ are the shear moduli of the Maxwell body and Kelvin body respectively, *η*_M_ and *η*_K_ are the viscosity coefficients of the Maxwell body and Kelvin body respectively. The model can characterize the instantaneous elastic deformation, delayed elastic deformation, and viscous flow behavior of surrounding rock. As a result, the model is well suited for describing the long-term deformation characteristics of soft rock surrounding rock.

Based on physical and mechanical derivation, the following one-dimensional creep equation of the Burgers constitutive model can be obtained^27^.

1 $$\varepsilon \left( t \right)=\sigma \left[ {\frac{1}{{{E_{\mathrm{M}}}}}+\frac{1}{{{n_{\mathrm{M}}}}}t+\frac{1}{{{E_{\mathrm{K}}}}}\left( {1 - {e^{\frac{{{E_{\mathrm{K}}}}}{{{n_{\mathrm{K}}}}}t}}} \right)} \right]$$

Where *E*_M_ and *E*_K_ are the elastic moduli of the Maxwell body and Kelvin body respectively; *n*_M_ and *n*_K_ are the viscosity coefficients of the Maxwell body and Kelvin body under one-dimensional state respectively; and *t* is the creep deformation time of rock mass.

Based on the indoor uniaxial creep test data, the parameters of the Burgers model under one-dimensional state can be obtained by mathematical fitting according to Eq. (1). However, actual roadway surrounding rock is in a three-dimensional spatial stress state. Therefore, it is necessary to extend the model parameters to a three-dimensional state in order to better simulate the real environment. To address this issue, Sun Jun, academician of CAE member, proposed a method to extend one-dimensional Burgers model parameters to a three-dimensional creep model, which only requires parameter conversion according to the following equations^28^.

2 $$K=\frac{{{E_{\mathrm{M}}}}}{{3\left( {1 - 2\mu } \right)}};\begin{array}{*{20}{c}} {} \end{array}\begin{array}{*{20}{c}} {} \end{array}{G_{\mathrm{M}}}=\frac{{{E_{\mathrm{M}}}}}{{2\left( {1+\mu } \right)}};\begin{array}{*{20}{c}} {} \end{array}\begin{array}{*{20}{c}} {} \end{array}{G_{\mathrm{K}}}=\frac{{{E_{\mathrm{K}}}}}{3};\begin{array}{*{20}{c}} {} \end{array}\begin{array}{*{20}{c}} {} \end{array}{n_{\mathrm{M}}}=\frac{{{\eta _{\mathrm{M}}}}}{3};\begin{array}{*{20}{c}} {} \end{array}\begin{array}{*{20}{c}} {} \end{array}{n_{\mathrm{K}}}=\frac{{{\eta _{\mathrm{K}}}}}{3};$$

Wher *µ* is Poisson’s ratio of rock mass.

Rock samples were collected from Banji Coal Mine and prepared into standard test specimens of coal-rock mass. After creep testing, data fitting, parameter conversion, and other steps, the Burgers creep model parameters for the roadway coal-rock mass were obtained as shown in Table 3.

**Table 3:** Mechanical properties of coal rock.

Rock type	K (GPa)	G_M_ (Pa)	Ƞ_M_ (Pa·s)	G_K_ (Pa)	Ƞ_K_ (Pa·s)
Sandy mudstone	3.37	1.73 × 10⁹	5.0 × 10¹⁶	0.86 × 10⁹	3.0 × 10¹⁶
No.6 coal seam	1.13	0.58 × 10⁹	1.0 × 10¹⁵	0.29 × 10⁹	5.0 × 10¹⁴
Mudstone	0.86	0.42 × 10⁹	1.0 × 10¹⁵	0.21 × 10⁹	2.7 × 10¹⁴
Fine sandstone	6.44	3.31 × 10⁹	1.0 × 10¹⁸	1.65 × 10⁹	5.0 × 10¹⁷

### Stability analysis of surrounding rock under original support

After the model reached initial stress equilibrium, the numerical simulation was conducted using step-by-step roadway excavation, with an excavation advance of 1 m per step, and support conditions were applied synchronously. Upon completion of roadway excavation, a creep calculation with a duration of three months was performed.

(1) Surrounding rock stress analysis.

**Fig. 12:**
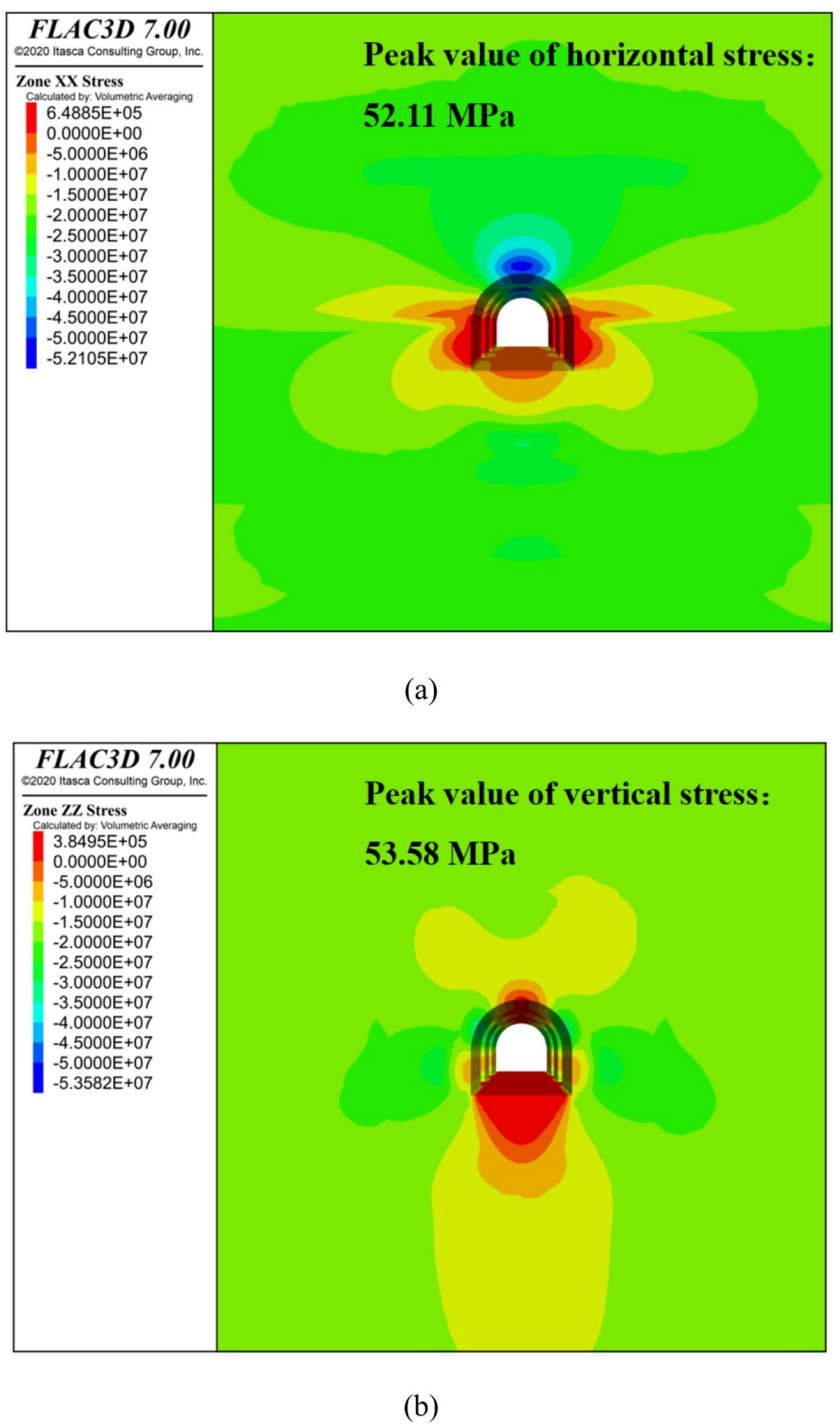
Stress cloud distribution of surrounding rock. (a) Horizontal stress, (b) Vertical stress.

Following excavation of the machine head section roadway, the stress field was significantly redistributed, as illustrated in Fig. 12. A pronounced horizontal stress concentration zone developed within the roof strata, with a peak value of 52.11 MPa. In contrast, a marked vertical stress concentration occurred along the inner wall of the machine head chamber, reaching a peak value of 53.58 MPa. Compared with these regions, the floor strata, owing to their weaker lithology and lower load-bearing capacity, were unable to sustain stress accumulation and predominantly exhibited stress release during redistribution. This stress release reduced floor stability and rendered the floor more susceptible to floor heave failure.

(2) Surrounding rock displacement analysis.

**Fig. 13:**
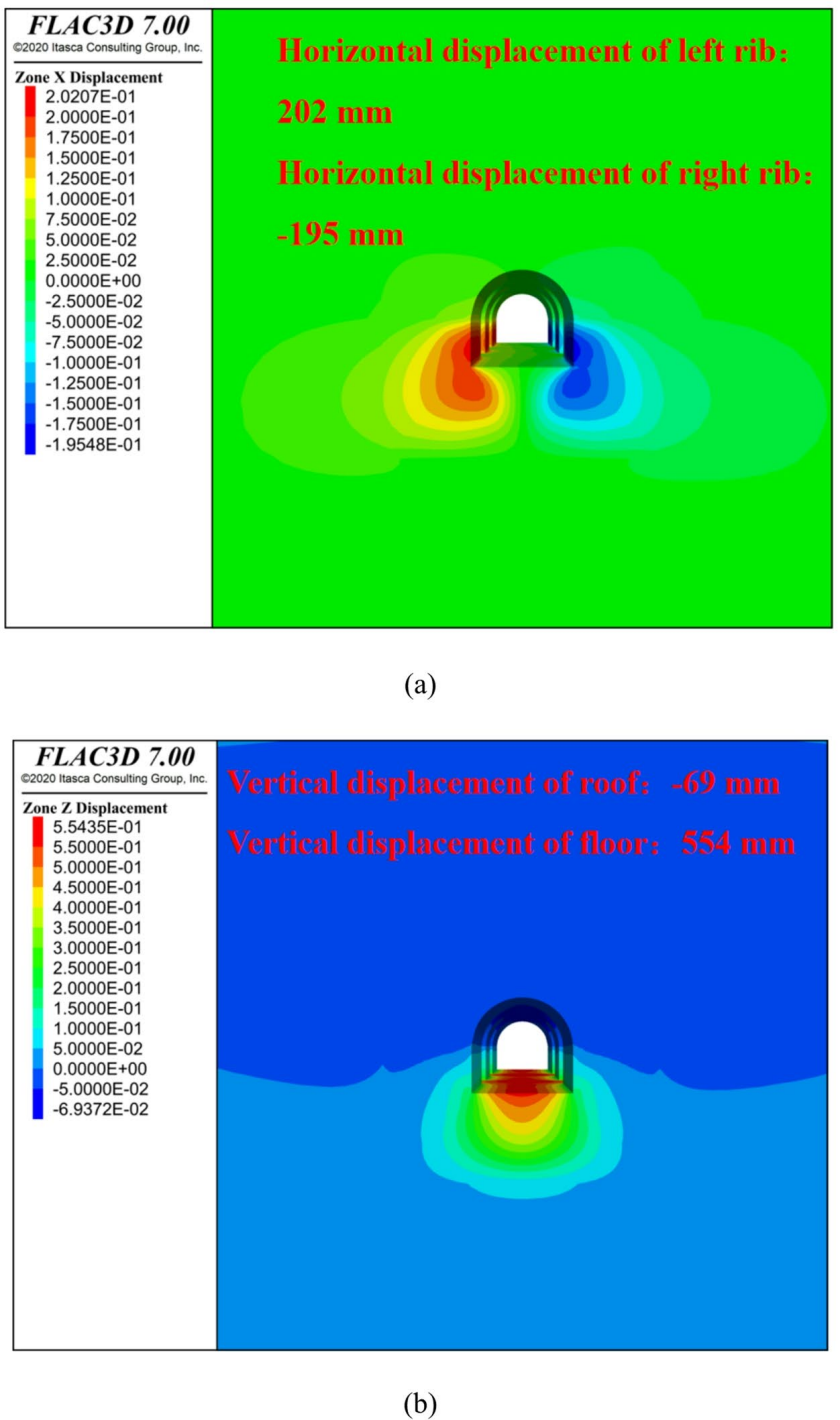
Displacement cloud distribution of surrounding rock. (a) Horizontal displacement, (b) Vertical displacement.

After excavation of the machine head section roadway and subsequent three-month creep evolution, the deformation of the surrounding rock increased markedly, as shown in Fig. 13. The roadway roof experienced a subsidence of 69 mm, the floor exhibited a heave of 554 mm, and the total convergence of the two ribs reached 397 mm. Comparative analysis indicates that deformation of the floor and ribs was substantially greater than that of the roof, suggesting that these zones were subjected to higher deformation demand and were characterized by relatively weaker lithology. This deformation pattern demonstrates the necessity of further strengthening and optimizing the support system for the floor and ribs to enhance their stability and resistance to deformation.

### Stability analysis of surrounding rock after reinforcement support

Based on the original support scheme, the roadway support system was further reinforced, and FLAC3D numerical simulation was employed to evaluate the stability of the surrounding rock under reinforced conditions. The main purposes of floor surrounding rock grouting reinforcing are to drain floor water and improve the mechanical strength of surrounding rock, with the focus on strengthening the mudstone layer that has relatively weak strength, high degree of fragmentation, and is prone to weakening when water-soaked. So, the reinforcement effect of the integrated deep-hole high-pressure grouting and drainage technique can be represented by increasing the mechanical parameters of the floor shale. The parameter settings are listed in Table 4. The mechanical parameters of shale after reinforcement were determined by combining laboratory test results with the original rock parameters.

**Table 4:** Mechanical parameters of the floor surrounding rock after grouting reinforcement.

Rock type	Weight (kN /m^3)^	Elastic modulus(GPa)	Shear modulus(GPa)	Internal friction angle(degree)	Cohesion (MPa)	Poisson’s ratio
Mudstone	2461	4.83	1.87	29	4.8	0.29

(1) Surrounding rock stress analysis.

**Fig. 14:**
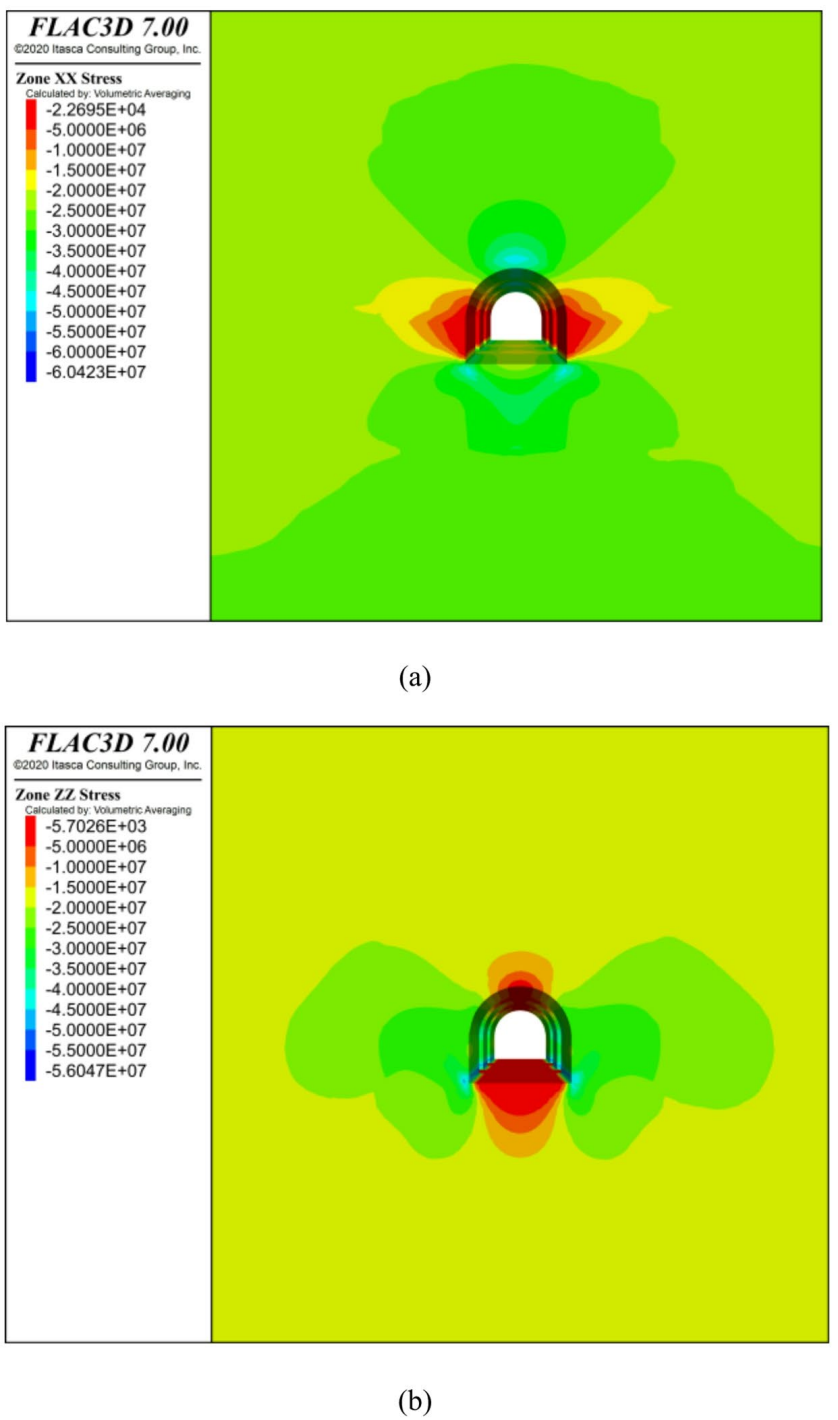
Stress cloud distribution of surrounding rock after reinforcement support. (a) Horizontal stress, (b) Vertical stress.

Figure 14 below illustrates the stress distribution of the surrounding rock after grouting reinforcement support under the proposed scheme.

It can be observed that, following reinforcement on the basis of the original support system in the machine head section roadway, the surrounding rock stress distribution was markedly improved. Stress was gradually transferred to deeper regions, and the extent of stress concentration was effectively reduced. Owing to the enhanced load-bearing capacity f the floor after grouting reinforcement, a certain degree of stress concentration occurred at the floor corners. Meanwhile, part of the surrounding rock stress was transferred to rock bolts and cables, which further alleviated stress concentration, with stress levels remaining within a controllable range.

(2) Surrounding rock displacement analysis.

**Fig. 15:**
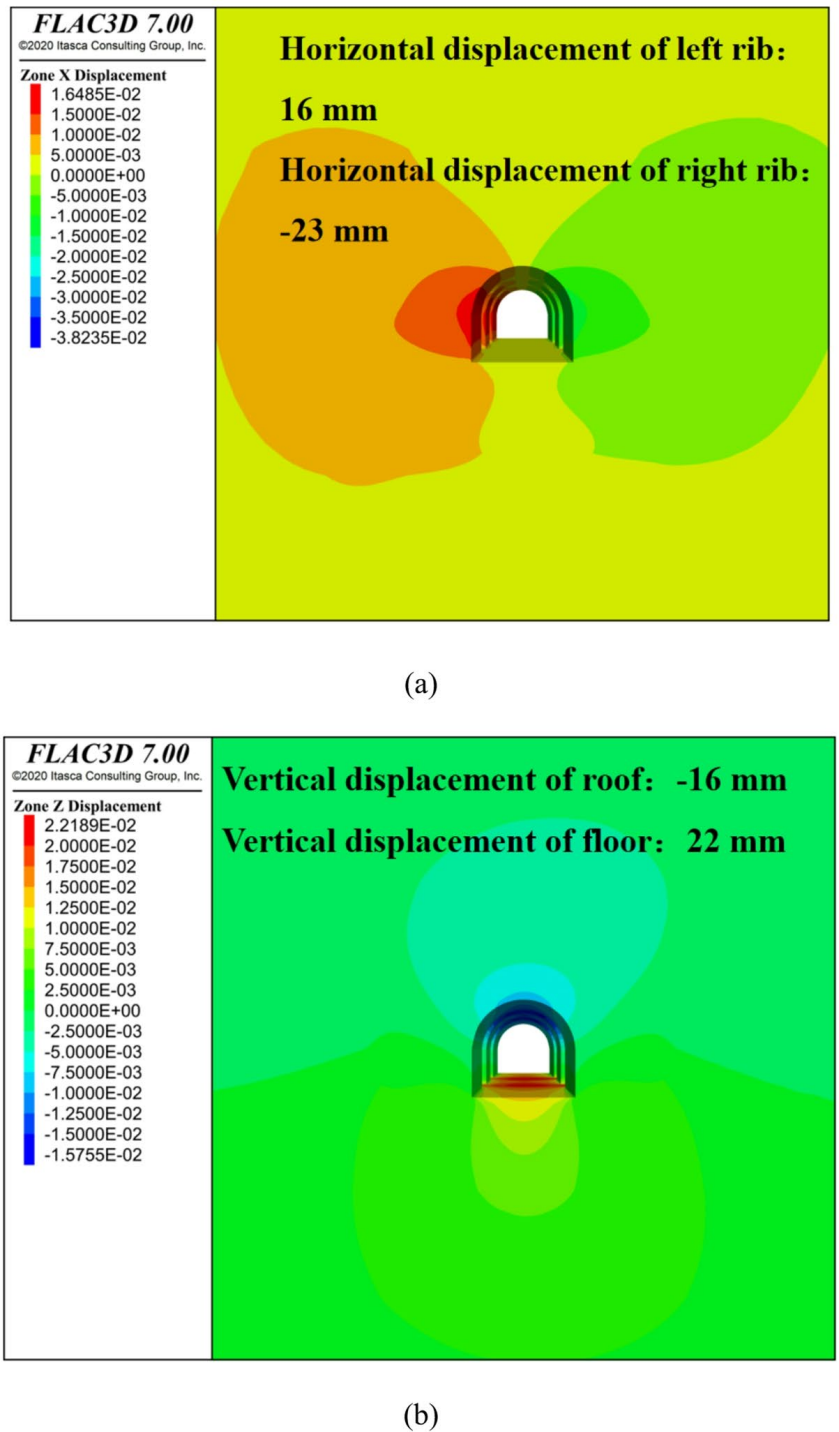
below presents the displacement distribution of the surrounding rock after grouting reinforcement support under the proposed scheme.

Figure 15 Displacement cloud distribution of surrounding rock after reinforcement support. (a) Horizontal displacement, (b) Vertical displacement.

Compared with the original support condition, the surrounding rock displacement of the machine head section roadway was significantly reduced under the reinforced support scheme. Under the reinforced condition, roof subsidence was 16 mm, floor heave was 22 mm, and the total convergence of the two ribs was 39 mm. Relative to the original support scheme, these values were reduced by 76.8%, 96.0%, and 90.2%, respectively. These results demonstrate that the reinforced support scheme effectively suppresses surrounding rock deformation and significantly enhances the structural stability of the machine head section roadway.

### Comparative analysis of reinforcement support effect

**Fig. 16:**
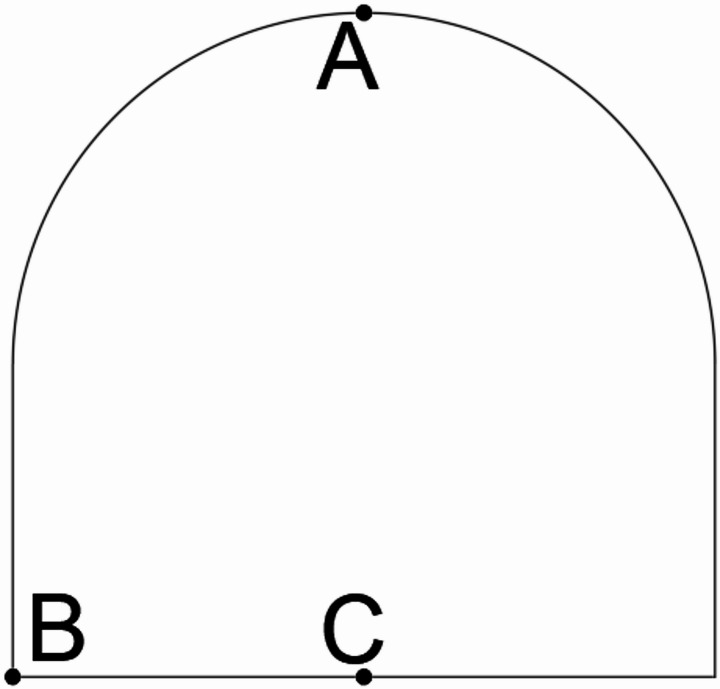
Displacement monitoring points.

To provide an intuitive verification of the control effect of the reinforced support scheme on floor heave, the middle section of the machine head section roadway between the two chambers was selected as the monitoring zone. Three displacement monitoring points, denoted as A, B, and C, were arranged at this section, as illustrated in Fig. 16.

**Fig. 17:**
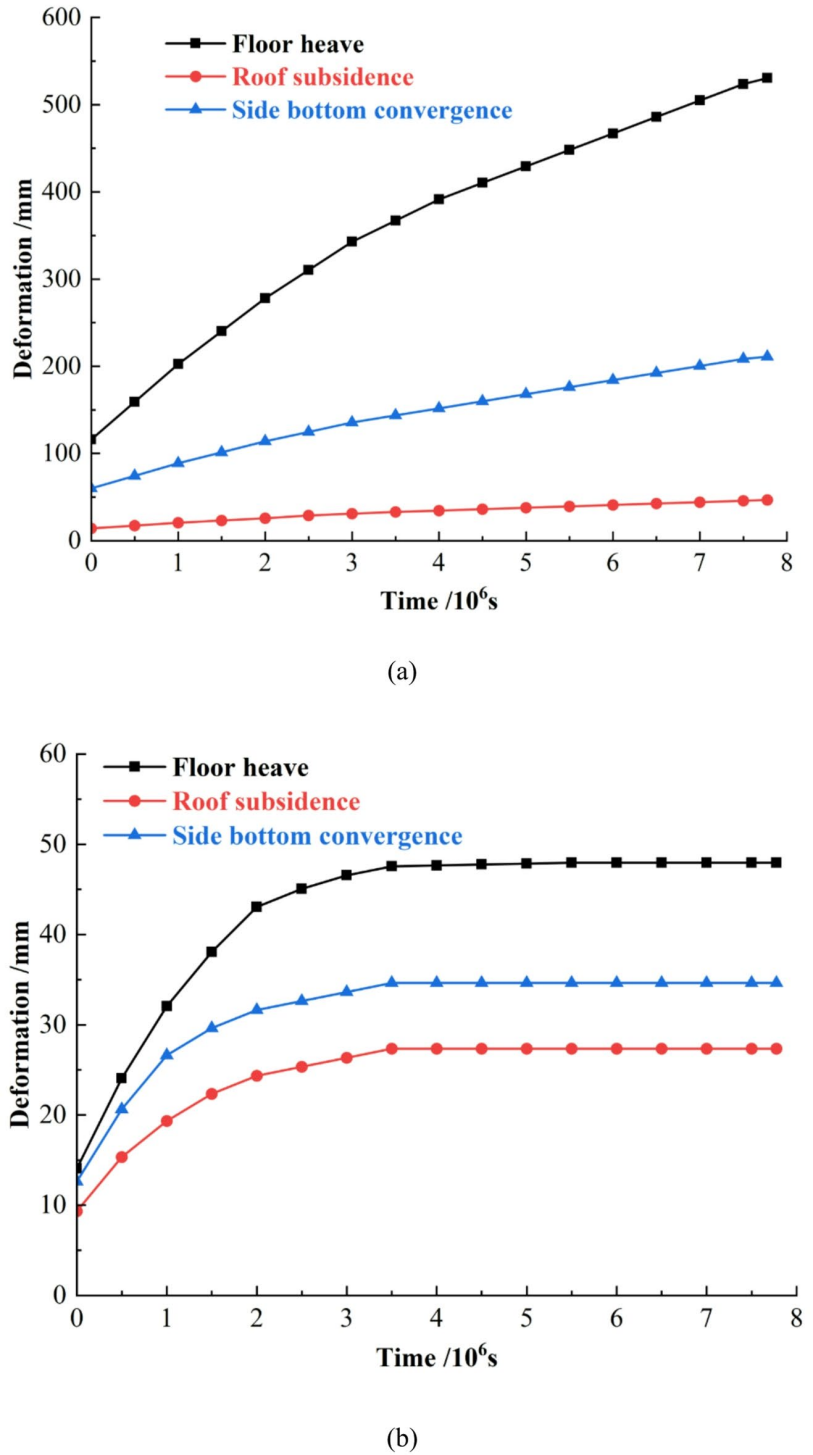
presents the displacement-time curves of the three monitoring points before and after grouting reinforcement support.

Figure 17 Displacement-time curves of three monitoring points before and after reinforcement support. (a) Before grouting reinforcement support, (b) After grouting reinforcement support.

As shown in Fig. 17, prior to grouting, the displacement at each key monitoring point increased continuously with creep time. Among them, the floor monitoring point C exhibited the largest cumulative deformation, reaching the order of hundreds of millimeters, and its displacement curve displayed pronounced creep characteristics. From the perspective of curve morphology, the surrounding rock deformation increases rapidly in the initial stage, and then the growth rate gradually slows down, exhibiting typical characteristics of transition from the decelerating creep stage (the first stage) to the steady creep stage (the second stage); no phenomenon of continuously increasing deformation rate occurs within the calculation period, indicating that the surrounding rock has not yet entered the accelerating creep stage (the third stage). With the progression of creep, although the growth rate of floor displacement gradually decreased, deformation continued to develop, indicating that floor heave was difficult to control effectively under the original support condition.

After grouting reinforcement, displacement levels at all key monitoring points were significantly reduced. The cumulative deformation of the floor decreased to the order of tens of millimeters, and the displacement curves exhibited an overall gentle trend, with no evident accelerated deformation stage during the later creep period. Compared with the displacement behavior prior to reinforcement, grouting reinforcement effectively reduced both the creep rate and cumulative deformation of the floor, thereby markedly suppressing the sustained development of floor heave. Meanwhile, following effective control of floor heave, the overall stress state of the surrounding rock was improved, and deformation of the roof and ribs wassynchronously alleviated. These monitoring results verify the effectiveness of the reinforced support scheme in controlling long-term deformation of the roadway.

## Field measurement

**Fig. 18:**
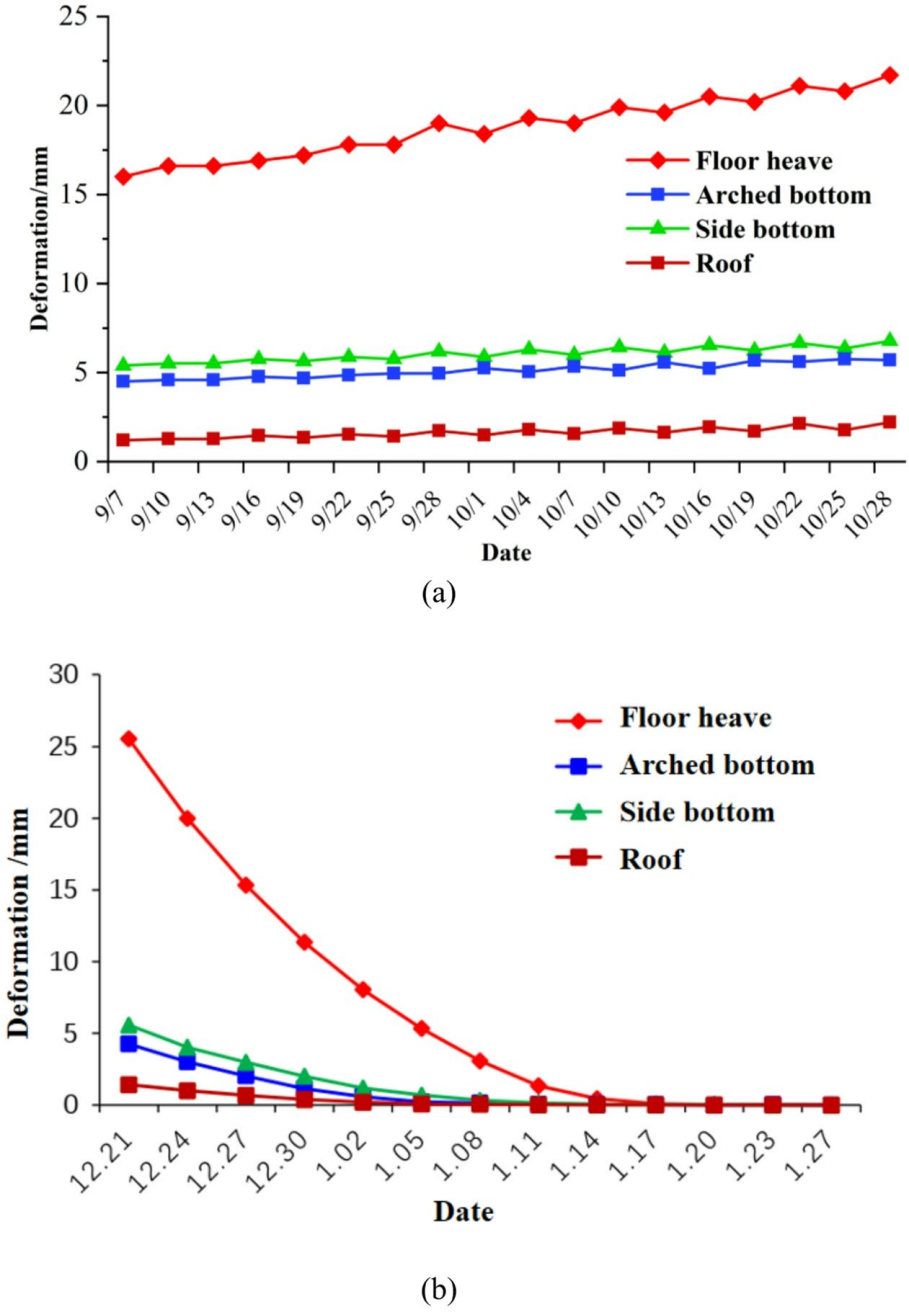
Deformation convergence curves of surrounding rock after grouting. (**a**) Before grouting, (**b**) After grouting.

To verify the reinforcement effect of grouting and evaluate the rationality of the proposed technical solution, deformation monitoring was conducted at the middle section of the grouting reinforcement zone. The monitoring indicators included the convergence rate of roof subsidence, the convergence rate between the two arch bases, the convergence rate of the two sides at the bottom, and the convergence rate of floor heave at the monitoring section. The monitoring results are presented in Fig. 18.

As indicated by the deformation convergence rate curves in Fig. 18, before grouting construction, the roadway surrounding rock deformation was significant and showed a certain trend of intensification. While 30 d after grouting, the subsidence of the roadway floor, the inward convergence of the two sides, and the floor heave were all basically stable. This deformation stabilization behavior is generally consistent with the numerical simulation results, further confirming the effectiveness of the grouting reinforcement scheme.

## Discussion

Owing to the extremely complex geological and engineering conditions of underground coal mine projects, together with the limitations of existing theoretical approaches, the mechanisms and causes of floor heave in deep soft rock roadways exhibit significant diversity. Consequently, corresponding reinforcement and control measures generally lack strong universality. The multifactorial disaster-causing mechanism and control technology for floor heave in deep soft rock roadways presented in this paper. Through grouting solidification and other means, the floor surrounding rock can be reinforced and effectively integrated with the existing floor and sidewall support structures, thereby forming an integrated collaborative control structure to control roadway deformation. However, this technology is derived from a case-specific investigation of the machine head section of the belt conveyor downhill roadway in the 1105 mining area of Banji Coal Mine and is therefore applicable primarily to roadway floor heave control under similar engineering and geological conditions. It has currently been implemented on-site in multiple mines in Southwest China, including Juxin Coal Mine, Panzhihua Coal Mine, Tengqing Coal Mine, Quanlun Coal Mine, Nazuo Coal Mine, and Sijichun Coal Mine, with significant effects on floor deformation control. Nevertheless, the research methods adopted and the underlying principles for support scheme design presented in this study possess certain theoretical value and provide practical reference for related engineering applications.

## Conclusions

Based on the engineering geological conditions and surrounding rock deformation characteristics of the machine head section of the belt conveyor downhill roadway in the 1105 mining area of Banji Coal Mine, the primary causes of severe floor heave were systematically analyzed. On the basis of on-site loosening zone test results, a “deep-hole high-pressure grouting and draining integration” reinforcement support technology was designed and implemented, leading to the following conclusions.

(1) Under the combined influence of deep high-stress conditions and intense mining activities, floor heave in the machine head section roadway is mainly attributed to the high-stress environment, the coupling effect between roadway cross-sectional geometry and surrounding rock stress distribution, the poor strength properties of the floor strata, as well as insufficient initial support capacity and uncoordinated deformation of the surrounding rock.

(2) The effectiveness of the reinforcement support measures in controlling surrounding rock deformation was verified through a combination of numerical simulation and on-site industrial testing. The results indicate that the proposed reinforcement support technology can effectively suppress roadway deformation and satisfy engineering stability requirements, thereby providing a reliable technical reference for roadway support under similar geological conditions.

## Data Availability

The data that support the findings of this study are available from the corresponding author upon reasonable request.
